# CBCT-based online adaptive radiotherapy of the prostate bed: first clinical experience and comparison to nonadaptive conventional IGRT

**DOI:** 10.1007/s00066-024-02323-6

**Published:** 2024-11-05

**Authors:** J. Fischer, L. A. Fischer, J. Bensberg, N. Bojko, M. Bouabdallaoui, J. Frohn, P. Hüttenrauch, K. Tegeler, D. Wagner, A. Wenzel, D. Schmitt, M. Guhlich, M. Leu, R. El Shafie, G. Stamm, A.-F. Schilling, L. H. Dröge, S. Rieken

**Affiliations:** 1 Department of Radiotherapy and Radiation Oncology, University Medical Center Göttingen, Göttingen, Germany; 2 Comprehensive Cancer Center, University Medical Center Göttingen, Göttingen, Germany; 3 Institute for Diagnostic and Interventional Radiology, University Medical Center Göttingen, Göttingen, Germany; 4 Department of Trauma Surgery, Orthopedics and Plastic Surgery, University Medical Center Göttingen, Göttingen, Germany

**Keywords:** Image-guided radiotherapy, Intensity-modulated radiotherapy, Prostate cancer, Target coverage, Organs at risk

## Abstract

**Purpose:**

Conventional image-guided radiotherapy (IGRT) of the prostate bed is challenged by the varying anatomy due to dynamic changes of surrounding organs such as the bladder and rectum. This leads to changed dose coverage of target and surrounding tissue. The novel online adaptive radiotherapy (oART) aims to improve target coverage as well as reduce dose exposure to surrounding healthy tissues by daily reoptimization of treatment plans. Here we set out to quantify the resulting changes of this adaptation for patients and treatment team.

**Methods:**

A total of 198 fractions of radiotherapy of the prostate bed (6 patients) were treated using oART with the Ethos accelerator (Varian Medical Systems, Palo Alto, CA, USA). For each fraction, volumes and several dose–volume parameters of target volumes and organs at risk were recorded for the scheduled plan (initial plan, recalculated based on daily cone beam computed tomography [CBCT]), the adapted plan, and the verification plan, which is the dose distribution of the applied plan recalculated on the closing CBCT after the adaptation process. Clinical acceptability for all plans was determined using given dose–volume parameters of target volumes. Additionally, the time needed for the adaptation process was registered and compared to the time required for the daily treatment of five conventional IGRT patients.

**Results:**

Volumes of target and organs at risk (OAR) exhibited broad variation from day to day. The differences in dose coverage D_98%_ of the clinical target volume (CTV) were significant through adaptation (*p* < 0.0001; median D_98%_ 97.1–98.0%) and further after verification CBCT (*p* < 0.001; median D_98%_ 98.1%). Similarly, differences in D_98%_ of the planning target volume (PTV) were significant with adaptation (*p* < 0.0001; median D_98%_ 91.8–96.5%) and after verification CBCT (*p* < 0.001; median D_98%_ 96.4%) with decreasing interquartile ranges (IQR). Dose to OAR varied extensively and did not show a consistent benefit from oART but decreased in IQR. Clinical acceptability increased significantly from 19.2% for scheduled plans to 76.8% for adapted plans and decreased to 70.7% for verification plans. The scheduled plan was never chosen for treatment. The median time needed for oART was 25 min compared to 8 min for IGRT.

**Conclusion:**

Target dose coverage was significantly improved using oART. IQR decreased for target coverage as well as OAR doses indicating higher repeatability of dose delivery using oART. Differences in doses after verification CBCT for targets as well as OAR were significant compared to adapted plans but did not offset the overall dosimetric gain of oART. The median time required is three times higher for oART compared to IGRT.

**Supplementary Information:**

The online version of this article (10.1007/s00066-024-02323-6) contains supplementary material, which is available to authorized users.

## Introduction

Adaptive radiotherapy enables the adaptation of radiation plans based on up-to-date anatomy just prior to application to a patient [1]. Daily imaging followed by patient realignment and the application of a predesigned treatment plan (image guided radiotherapy [IGRT]) may suffice to deliver an adequate dose for certain body regions. In areas with relevant organ movement, such as the pelvic region’s bowel or bladder, IGRT may result in underdosing the target or overexposing surrounding healthy tissues. IGRT can only account for interfractional translational and possibly rotational variations, not the changing shape and size of organs and tumors [2]. Traditionally, this is addressed by incorporating sufficient margins around the clinical target volume (CTV) to accommodate potential deviations, thereby, ensuring that the planning target volume (PTV) encompasses the intended treatment area, thus, guaranteeing adequate target coverage to the CTV [3, 4].

Online adaptive radiotherapy (oART) emerges as a technique to address highly variable treatment sites by reoptimizing the predesigned treatment plan to fit the actual anatomy, while the patient is located in the treatment room [5]. However, creation of radiotherapy plans is time-consuming and involves various professionals like physicians and medical physicists. To facilitate timely plan creation, this staff must be present during treatment, and processes need acceleration and automation [6].

Progress in artificial intelligence (AI) coupled with increased computational power have led to initial commercial solutions for oART [7]. These solutions include automated deformable image registration algorithms, automated segmentation, and computer-based independent dose recalculation [8]. However, limited data exist regarding which patients or types of cancer, respectively, benefit most from oART and the practical demands in terms of additional personnel or time [9].

We present our initial experiences with computed tomography (CT)-based online adaptive radiotherapy, focusing on prostate bed treatments. For these treatment areas, interfractional motion is well known and has been investigated using several technical approaches [10, 11]. In the present study, dose distributions of adapted and conventional treatments and the additional time required for oART compared to conventional IGRT were evaluated.

## Materials, patients, and methods

### Study design and patient selection

The Ethos system (Varian Medical Systems, Palo Alto, CA, USA) is a ring-gantry-based linear accelerator that offers the capability of AI-driven kV-cone-beam-CT-based oART. It was installed at the University Medical Center Göttingen in August 2022. Initially, only IGRT was utilized in clinical settings. However, starting from January 2023, the integration of oART into routine practice commenced. At first, prostate bed treatments were chosen for oART, with the treatment of other types of cancer at a later stage. For the purpose of this analysis, we focused on the first 198 oART fractions administered to the first consecutive 6 patients for prostate bed treatments. Furthermore, 5 conventional IGRT patients with simultaneous treatment initiation were included into the analysis to compare the time need per fraction of oART to conventional IGRT technique. All patients included in this analysis received a cumulative dose of 66 Gy delivered in 33 fractions to the prostate bed (CTV), with a 5 mm margin (PTV). This analysis was approved by the local ethics committee (no. 7/8/23). The study was conducted in accordance with the Declaration of Helsinki.

### Workflow description

**Fig. 1:**
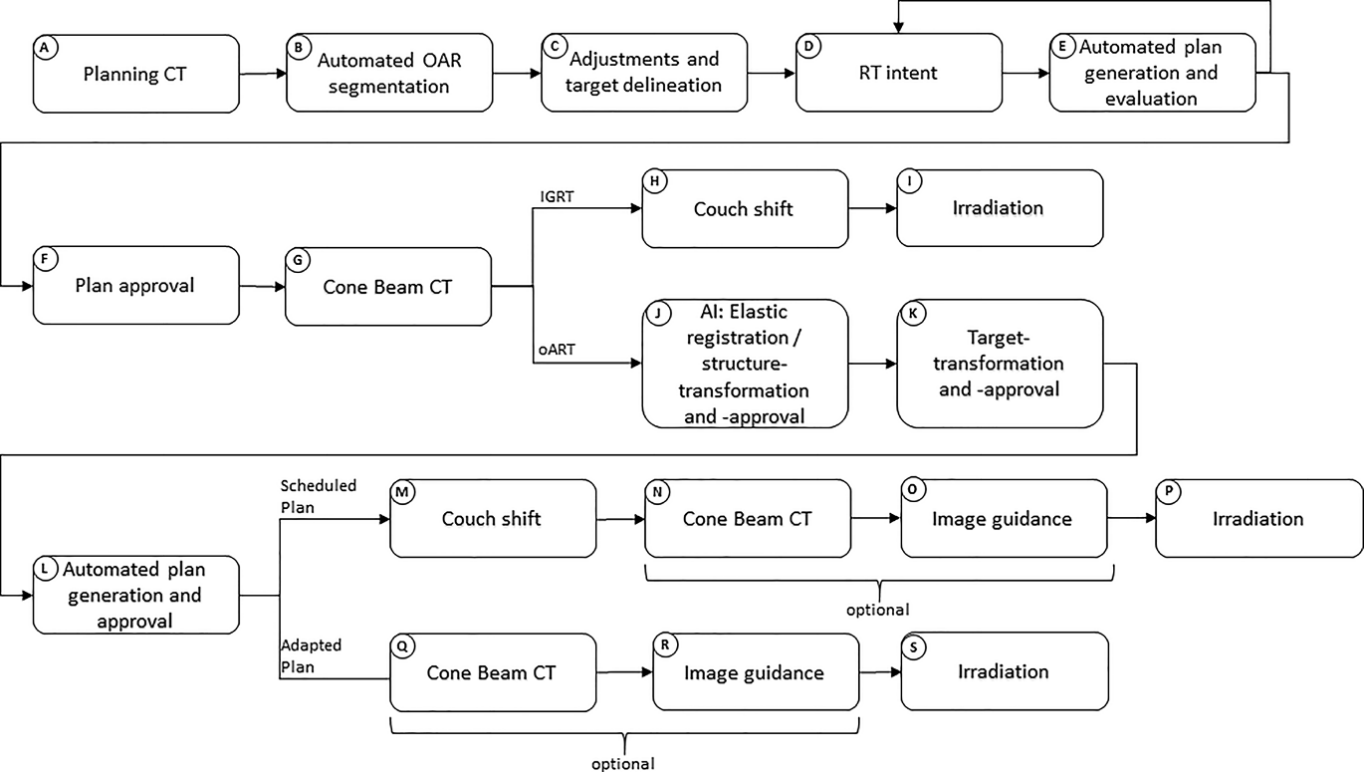
The flow chart shows the process of online adaptive radiotherapy (oART) and image-guided radiotherapy (IGRT), respectively, using Ethos, from initial planning computed tomography (CT) to irradiation. *AI* artificial intelligence

Figure 1 illustrates the workflow for oART and IGRT treatment planning and delivery, as well as the data acquisition process for this analysis.

Following the planning CT (pCT) {A}, automated segmentation of Organs at Risk (OARs) was performed {B} using AI-based auto-contouring software (Limbus Contour, v 1.8.0-B3, Limbus AI Inc., Regina, SK, Canada). Adjustments, if needed, and target delineation were carried out manually by physicians {C}. Within the Ethos treatment planning software (v 2.1), an RT intent was generated {D}, containing all information regarding fractionation, dosage, whether or not to adapt, and the intended maximum exposure of OARs in terms of dose–volume constraints. Ethos then generated a selection of multiple treatment plans (intensity-modulated radiation therapy [IMRT] and volumetric modulated arc therapy [VMAT]) {E} from which one had to be chosen for treatment. If no satisfactory plan was proposed, steps {D} (with adjustments) and {E} were iteratively repeated until an acceptable treatment plan was found and chosen for approval {F}.

Each treatment session began with the acquisition of a cone-beam CT (CBCT) {G}. For IGRT treatment, a conventional workflow involving couch shifting {H} was performed before irradiation {I}. In contrast, for oART, an automated deformable image registration of the initial pCT to the actual CBCT was performed and a resulting synthetic CT (sCT) was created by the system. This sCT contained the Hounsfield values from the initial pCT and the geometry of the actual CBCT. Thus, it was used for dose calculation in oART but is, for simplicity, hidden for the user. Followed by that, AI-driven OAR delineation with the option for manual corrections {J} was done. Once OARs were approved, initial targets were automatically and deformable transformed to the CBCT {K}, using registration of the pCT, with the possibility of manual corrections. Subsequently, two treatment plans were calculated and presented {L}: the scheduled plan (SCH), which was the initial plan recalculated on the sCT, incorporating an automatically determined couch shift {M}; and the adapted plan (ADP), a newly optimized plan derived from the initial plan’s geometry and specifications from {D}. After selection of one of the plans by a physician, performance of a secondary dose calculation in place of verification measurement (Mobius3D v 4.0.2, Varian Medical Systems, Palo Alto, CA, USA) and plan approval by a medical physicist, an additional verification CBCT {N} or {Q} with a conventional IGRT couch shift {O} or {R} (optional) was conducted to monitor potential patient shifts during the adaptation process. Therefore, the initial CBCT {G} was used as a reference. Finally, the patient underwent irradiation with either the SCH {P} or the ADP {S} plan. Subsequently, an automatic dose reconstruction of the applied plan on the verification CBCT was performed by the system (VER).

### Initial plan generation, endpoints, and statistics

All patients underwent a treatment planning procedure following the local standard clinical protocol including a pCT (Philips Brilliance Big Bore) with slice thickness of 3 mm. Patients were instructed to present with a comfortably filled bladder and emptied rectum for pCT and daily radiotherapy sessions. Therefore, patients received written instructions including advice for behavior in eating and drinking. Table 1 provides all the dose–volume specifications for the targets and OAR, which were used in the RT intent. Some patients may exhibit variant characteristics in anatomy, which means that intestinal loops could partially overlap with the PTV. To account for the dose specification D_0.1_ _cc_ (bowel) outlined in Table 1, the CTV-–PTV relationship was generally set as follows:

$$\mathrm{PTV}=\mathrm{CTV}\cup ((\mathrm{CTV}+5\,\mathrm{mm})-\text{Bowel})$$

**Table 1:** Recorded dose–volume histogram (DVH) parameter for target volumes and organs at risk

Organ	Dose–volume specification	Clinical standard
CTV	D_98%_	≥ 95%
PTV	D_98%_	≥ 95%
PTV	D_2%_	< 105%
Bladder	D_mean_	ALARA
Bladder	V_40Gy_	ALARA
Rectum	D_mean_	ALARA
Rectum	V_40Gy_	ALARA
Rectum	D_0.1_ _cc_	< 66 Gy
Bowel	D_0.1_ _cc_	< 60 Gy

*cc* cubic centimeter, *CTV* clinical target volume, *PTV* planning target volume, *ALARA* as low as reasonably achievable

This adjustment ensured that, in case of nearby bowel loops, the PTV was cropped by the region of overlap while still encompassing the CTV at a minimum.

All patients received either 9‑ or 12-field equally spaced IMRT, as VMAT was not established in clinical practice due to longer calculation and optimization time. This initial plan creation process was identical for both oART and IGRT.

The following parameters were recorded for analysis: all dose–volume histogram (DVH) parameters from Table 1, as well as the volumes of the CTV, PTV, bladder, and rectum. All volumes were normalized to the initial treatment plan to account for interfractional variations.

IGRT patients were treated using daily, CBCT-based image guidance. For oART patients a CBCT-based, new treatment plan, adapted to the actual anatomy was created for every fraction.

For IGRT patients, total treatment time (t_total, IGRT_: {G} to {I} in Fig. 1) was recorded for 10 fractions per patient (for simplicity), evenly distributed throughout the course of treatment. For every oART fraction, total treatment time (t_total, ART_: {G} to {P} or {S}, respectively, in Fig. 1) and time for adaptation (t_adapt_: {G} to {N} or {Q}, respectively) was recorded. Table 2 lists all time-related and volume parameters recorded.

**Table 2:** Recorded time-related and volume parameters

Parameter	Description	Source (Fig. 1)
T_total, IGRT_	Total time of treatment for IGRT (= time patient is opened to time patient is closed in software)	{G} to {P}
T_total, ART_	Total time of treatment for oART (= time patient is opened to time patient is closed in software)	{G} to {S}
T_adapt_	Time from first to second CBCT (= time for adaptive process)	{G} to {N} or {G} to {Q}
V_CTV_ V_PTV_ V_bladder_ V_rectum_	Volume of named structure	{J} and {K}

For all fractions, acceptability based on clinical standards for CTV and PTV (as shown in Table 1) was determined, meaning D_98%_ above 95% considered acceptable.

Comparative statistics regarding dose–volume parameters of targets or organs were performed using Wilcoxon signed-rank test, using patients as their own control. The null hypothesis was that there was no difference between parameters of the scheduled and adaptive plans, with a two-sided significance level at *p* < 0.05. Comparative statistics regarding treatment time were performed using Mann–Whitney U test. The null hypothesis was that time needs were not divergent, with a two-sided significance level at *p* < 0.05. Comparative statistics regarding acceptability of fractions were performed using McNemar test with null hypothesis declaring no difference in acceptability and significance level at *p* < 0.05. Comparative statistics regarding correlation of volumes were performed using Spearman correlation coefficient with null hypothesis being that there was no correlation. All statistics were computed using python (v3.12) with packages pandas (v2.2.1) and scipy (v1.12).

## Results

### Patient selection

This analysis included 6 patients with indications for irradiation of the prostate bed. All these patients received a total dose of 66 Gy in 33 fractions between January 2022 and September 2022. The median age of all patients in this study was 73 years (first quartile (Q1): 60.5; third quartile (Q3): 76.5). A total of 198 oART fractions were analyzed. In all these fractions, the adapted treatment plan was selected. The scheduled plan was never chosen due to poor target dose coverage and/or inferior dose to surrounding organs.

**Fig. 2:**
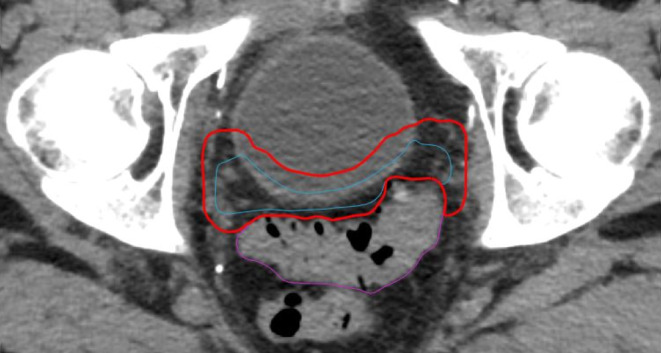
Patient 3—Delineation of the CTV (blue) and cropped PTV (red) influenced by the proximity of a nearby bowel loop (purple)

Patient 3 exhibited variant characteristics in anatomy where intestinal loops partially overlapped with the PTV. The CTV–PTV relationship described above lead to a cropped planning target volume, as shown in Fig. 2.

### Organs at risk

#### Volume differences

**Fig. 3:**
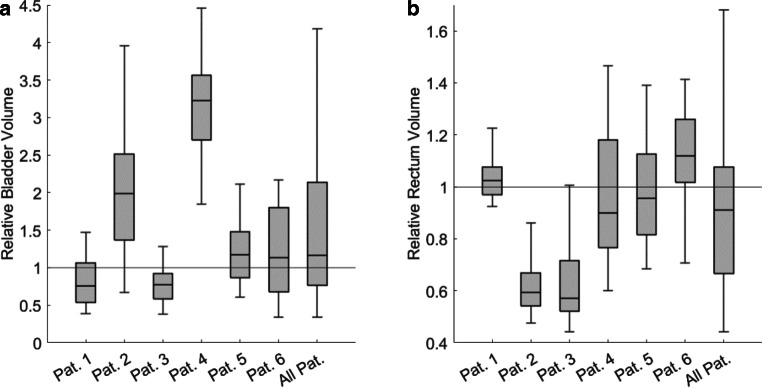
Relative volumes of the bladder (**a**) and rectum (**b**), each normalized to their initial volume in the planning computed tomography (CT), encompassing data for every individual patient (Pat.) as well as all patients collectively

The relative volumes of the bladder and rectum (normalized to its initial volume in pCT) exhibited variation during oART. Specifically, for the bladder, the median relative volume was 117% (Q1: 76; Q3: 213). In the case of the rectum, the median relative volume was 91% (Q1: 67; Q3: 108). These data are visualized in Fig. 3. The boxes illustrate the median, first and third quartiles, while the whiskers extend from the end of the interquartile range (IQR) to the furthest observation not deemed an outlier. Any data exceeding 1.5 times the IQR from the box’s border is considered an outlier. This definition applies to all box plots within this study.

#### Dose differences

**Fig. 4:**
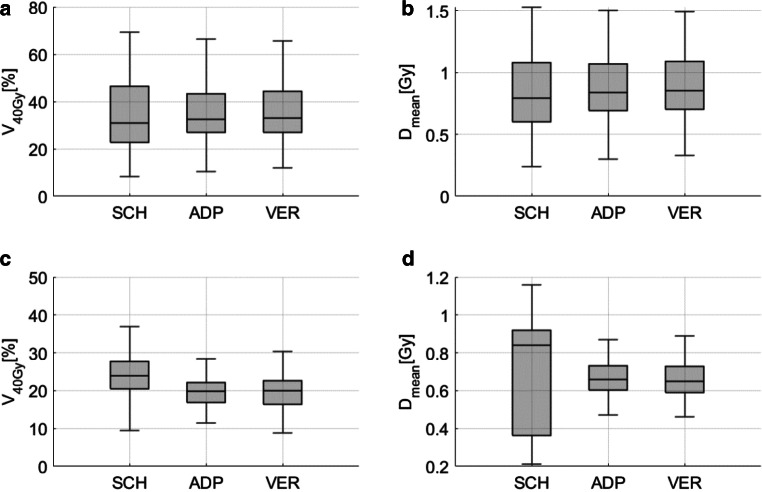
Dose exposure of bladder (**a**, **b**) and rectum (**c**, **d**) in terms of V_40Gy_ (**a**, **c**) and D_mean_ (**b**, **d**) for scheduled (SCH), adapted (ADP), and verification (VER) plans

Figure 4 depicts the dose exposure (in terms of D_mean_ and V_40Gy_) to the bladder and rectum for SCH, ADP, and VER plans across all patients. Regarding the bladder, a significant difference in V_40Gy_ was found between SCH and ADP (*p* < 0.001; median SCH: 31.0%; median ADP: 32.6%), as well as between SCH and VER (*p* < 0.001; median VER: 33.0%). For D_mean_, the differences between SCH and ADP (*p* < 0.001; median SCH: 0.80 Gy/fx; median ADP: 0.84 Gy/fx) as well as between SCH and VER (*p* < 0.001; median VER: 0.86 Gy/fx) were significant. The IQR (Q3 − Q1) decreased from SCH 23.7% to ADP 16.1% and VER 17.3% for V_40Gy_ and from SCH 0.48 Gy/fx to ADP 0.35 Gy/fx and VER 0.39 Gy/fx for D_mean_.

In the case of the rectum, D_mean_ was median 0.84 Gy/fx for SCH, 0.66 Gy/for ADP and 0.65 Gy/fx for VER without differences being significant, while differences in V_40Gy_ were significant between SCH and ADP (*p* < 0.0001; median SCH: 24.0%; median ADP: 19.8%) as well as between SCH and VER (*p* < 0.0001; median VER: 20.0%). The IQR decreased from SCH 0.56 Gy/fx to ADP 0.13 Gy/fx and to VER 0.14 Gy/fx for D_mean_ and from SCH 7.3% to ADP 5.2% and VER 6.2% for V_40Gy_.

**Fig. 5:**
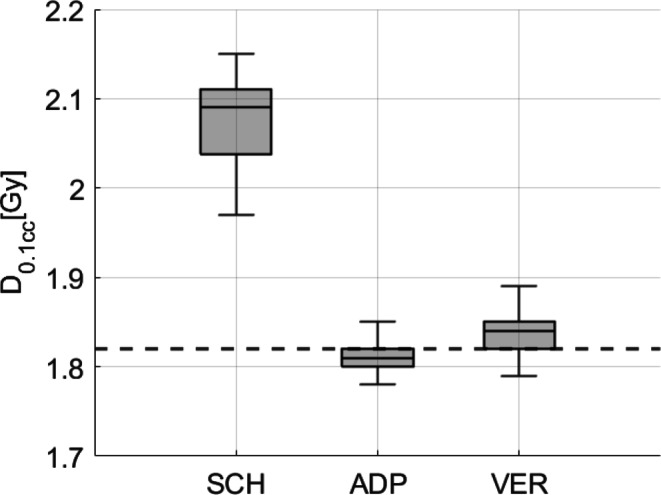
D_0.1_ _cc_ of the bowel for scheduled (SCH), adapted (ADP), and verification (VER) plans of patient 3. A dashed line marks the internal clinical standard constraint (60 Gy or 1.82 Gy/fx, respectively)

The relevance of bowel D_0.1_ _cc_ was minimal for 5 out of 6 patients, given a sufficient distance of the bowel from the target volume. For that reason bowel D_0.1_ _cc_ were not recorded or the bowel was not contoured for these patients. However, for patient 3, a deliberate underdosage of the PTV was applied to meet the specification of bowel D_0.1_ _cc_ < 60 Gy. As expected, this led to a substantial reduction in bowel D_0.1_ _cc_, decreasing from median 2.09 Gy/ fx for scheduled plans to 1.81 Gy/fx for adapted plans and to 1.84 Gy/fx in the verification dose, as shown in Fig. 5. IQR decreased from 0.07 Gy/fx for SCH to 0.02 Gy/fx for ADP and to 0.03 Gy/fx for VER. In this figure, the clinical standard constraint per fraction (60 Gy / 33 fx = 1.82 Gy/fx) is highlighted with a dashed line. All statistical data regarding the dose to OAR are provided in Table 3.

**Table 3:** The table presents statistical data for the bladder and rectum (V_40Gy_ and D_mean_) and bowel (D_0.1_ _cc_) for SCH, ADP, and VER plans

	Parameter	Plan	Median	Q1	Q3	IQR	*p*-value (to SCH)	*p*-value (to ADP)
Bladder	V_40Gy_	SCH	31.0%	22.8	46.5	23.7	–	–
		ADP	32.6%	27.1	43.2	16.1	< 0.001	–
		VER	33.0%	27.1	46.5	17.3	< 0.001	0.02
	D_mean_	SCH	0.80 Gy/fx	0.60	1.08	0.48	–	–
		ADP	0.84 Gy/fx	0.69	1.04	0.35	< 0.001	–
		VER	0.86 Gy/fx	0.70	1.09	0.39	< 0.001	< 0.001
Rectum	V_40Gy_	SCH	24.0%	20.4	27.7	7.3	–	–
		ADP	19.8%	16.9	22.1	5.2	< 0.0001	–
		VER	20.0%	16.4	22.6	6.2	< 0.0001	0.16
	D_mean_	SCH	0.84 Gy/fx	0.36	0.92	0.56	–	–
		ADP	0.66 Gy/fx	0.60	0.73	0.13	0.31	–
		VER	0.65 Gy/fx	0.59	0.73	0.14	0.16	0.07
Bowel patient 3	D_0.1_ _cc_	SCH	2.09 Gy/fx	2.04	2.11	0.07	–	–
		ADP	1.81 Gy/fx	1.80	1.82	0.02	< 0.001	–
		VER	1.84 Gy/fx	1.82	1.85	0.03	< 0.001	< 0.001

It includes the median, first quartile (Q1), third quartile (Q3), interquartile range (IQR, Q3–Q1), and *p*-values (Wilcoxon signed-rank test)

### Target volumes

#### Volume differences

**Fig. 6:**
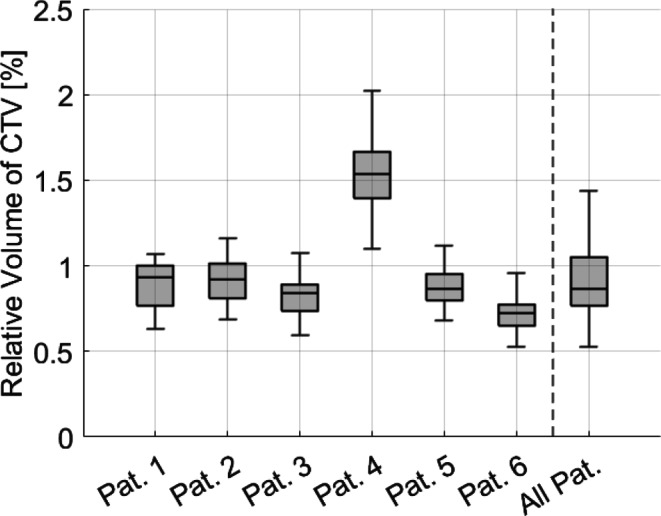
Relative volume of CTV, normalized to the initial volume in the planning CT, encompassing data for every individual patient (Pat.) as well as all patients collectively. With the exception of patient 4, all other patients exhibited decreased target volumes throughout the course of treatment

The volumes of the CTV and PTV were smaller during oART compared to the volumes obtained from the initial pCT. Specifically, the relative median volume for CTV was 86.7% (Q1: 76.7; Q3: 105.0), while for PTV, the relative median volume was 85.0% (Q1: 77.2; Q3: 99.6). In all patients, except for patient 4, the volumes of both CTV and PTV were lower during oART compared to the initial planning images. However, for patient 4, the relative median volume for CTV increased to 153.7% (Q1: 109.8; Q3: 202.4), and for PTV, it increased to 141.8% (Q1: 132.9; Q3: 149.0). A possible explanation will be discussed in the following section. The data for CTV is graphically presented in Fig. 6.

**Fig. 7:**
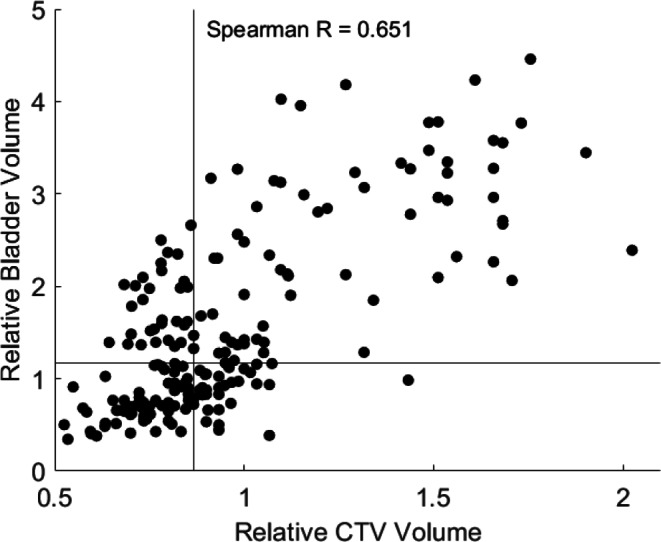
Scatter plot for CTV and bladder volume, Spearman R = 0.651. Horizontal and vertical lines represent the specific median volume

The Spearman correlation coefficient between volume of CTV and bladder was 0.651 (*p* < 0.0001), between volume of CTV and rectum it was 0.031 (*p* < 0.0001). Figure 7 shows the scatter plot for CTV and bladder volume.

#### Dose differences

**Fig. 8:**
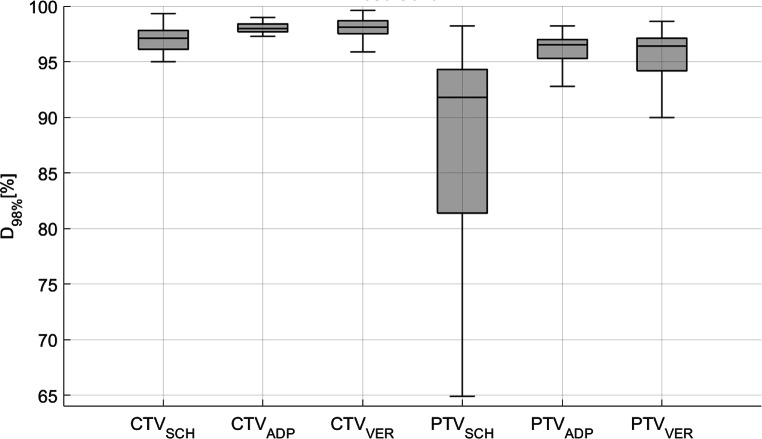
Dose coverage (D_98%_) for scheduled (SCH), adapted (ADP), and verification (VER) plans, implying significant improvement in differences in D_98%_ for CTV (left) as well as PTV (right)

Dose coverage of the CTV, as measured by D_98%_, was a median 97.1% for SCH plans, median 98.0% for ADP plans, and median 98.1% for VER. Regarding the PTV, D_98%_ was median 91.8% for SCH plans, median 96.5% for ADP plans, and median 96.4% for VER. These results are graphically presented in Fig. 8, indicating significant differences of dose coverage for both CTV (between SCH and ADP: *p* < 0.0001; between SCH and VER: *p* < 0.0001) and PTV (between SCH and ADP: *p* < 0.0001; between SCH and VER: *p* < 0.0001), along with a reduction in the IQR (from SCH to ADP and from SCH to VER).

**Fig. 9:**
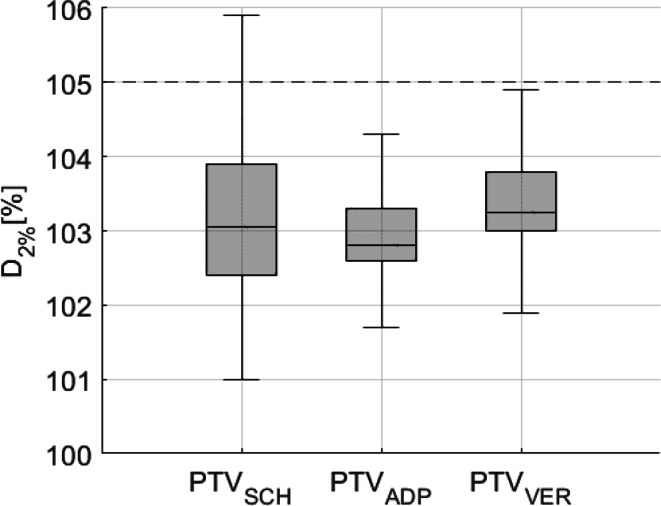
Planning target volume (PTV) near maximum dose (D_2%_) of PTV for scheduled (SCH), adapted (ADP), and verification (VER) plans exhibit a significant reduction in dose differences through adaptation. Internal clinical standard constraint (D_2%_ < 105%) is marked by a dashed line

For PTV near-maximum dose in terms of D_2%_, significant dose differences between SCH and ADP (*p* < 0.05; median SCH: 103.1%; median ADP: 102.8%) as well as between SCH and VER (*p* < 0.05; median 103.3%) were found, as shown in Fig. 9. In this figure, the clinical standard constraint (D_2%_ < 105%) is highlighted by a dashed line. The IQR decreased from SCH to ADP and to VER. All statistical data regarding the dose to targets are provided in Table 4.

**Table 4:** The table presents statistical data for the CTV and PTV (D_98%_ and D_2%_) for SCH, ADP, and VER plans

	Parameter	Plan	Median	Q1	Q3	IQR	*p*-value (to SCH)	*p*-value (to ADP)
CTV	D_98%_	SCH	97.1	96.1	97.8	1.7	–	–
		ADP	98.0	97.7	98.4	0.7	< 0.0001	–
		VER	98.1	97.5	98.7	1.2	< 0.0001	< 0.0001
PTV	D_98%_	SCH	91.8	81.4	94.3	12.9	–	–
		ADP	96.5	95.3	97.0	1.7	< 0.0001	–
		VER	96.4	94.2	97.1	2.9	< 0.0001	0.15
	D_2%_	SCH	103.1	102.4	103.9	1.5	–	–
		ADP	102.8	102.6	103.3	0.7	< 0.05	–
		VER	103.3	103.0	103.8	0.8	< 0.05	< 0.0001

It includes the median, first quartile (Q1), third quartile (Q3), interquartile range (IQR, Q3–Q1), and *p*-values (Wilcoxon signed-rank test)

**Fig. 10:**
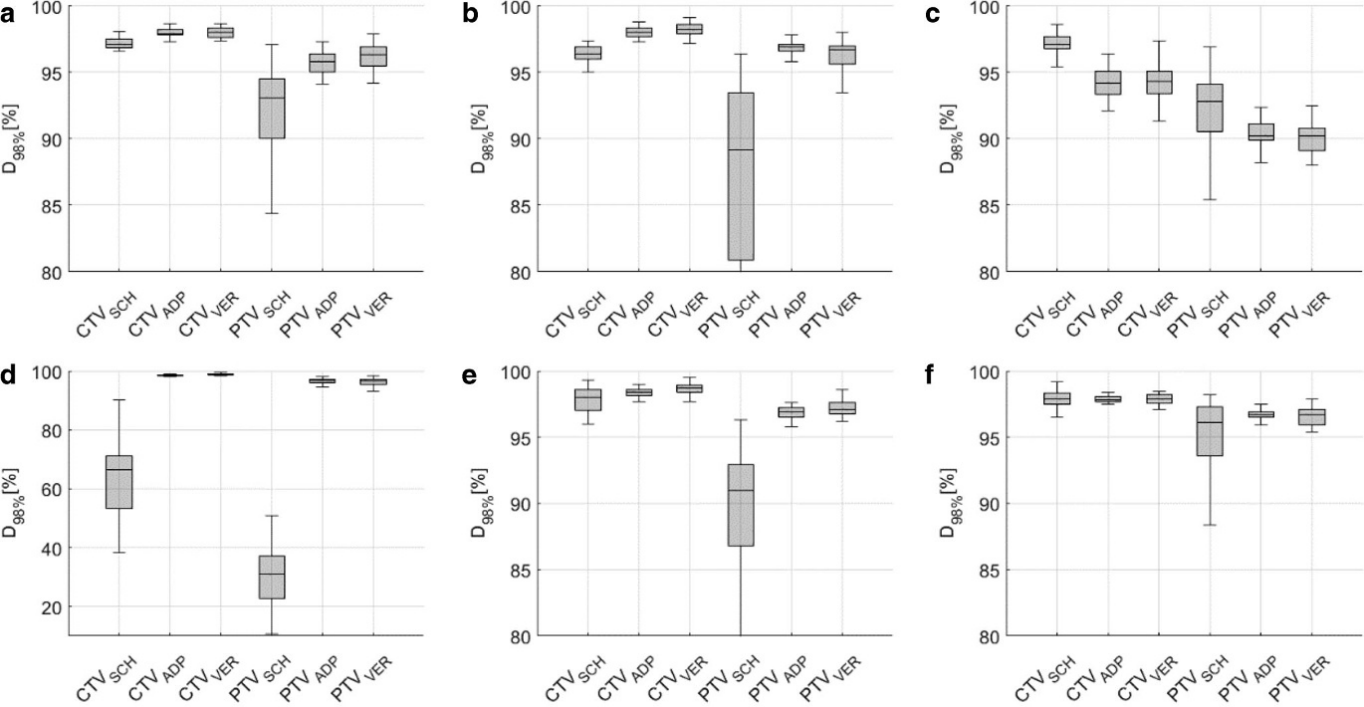
Dose coverage (D_98%_) of CTV (left in each subplot) and PTV (right in each subplot) for scheduled (SCH), adapted (ADP), and verification (VER) plans from patient 1 (**a**) to patient 6 (**f**) demonstrates a significant improvement for all patients except patient 3. The presence of an intestinal loop adjacent to the target volume in patient 3 necessitated adjustments to the target volume, resulting in a notable decrease in dose coverage. Please note the different scaling of the y‑axis in subplot 4 (**d**) for better readability

Figure 10 presents the resulting dose coverage for each individual patient. The variant characteristics of the nearby intestinal loop in patient 3 and the consequent adjustments made to the target volume resulted in a notable decrease in target coverage, as demonstrated in Fig. 10c. Simultaneously, this alteration led to a substantial reduction in bowel D_0.1_ _cc_ (as discussed in the previous section).

Considering D_98%_ of the CTV and PTV exceeding 95%, and D_2%_ of PTV remaining below 105% of the prescribed dose as the clinically acceptable range, 38 out of 198 fractions (19.2%) from SCH plans, 152 out of 198 fractions (76.8%) from ADP plans, and 140 out of 198 fractions (70.7%) from VER plans fell within this range. This demonstrates a strongly significant enhancement in plan acceptability from SCH to ADP as well as from SCH to VER (McNemar test, both *p* < 0.0001). However, from ADP to VER, a significant decline in acceptability was observed (*p* < 0.05).

**Fig. 11:**
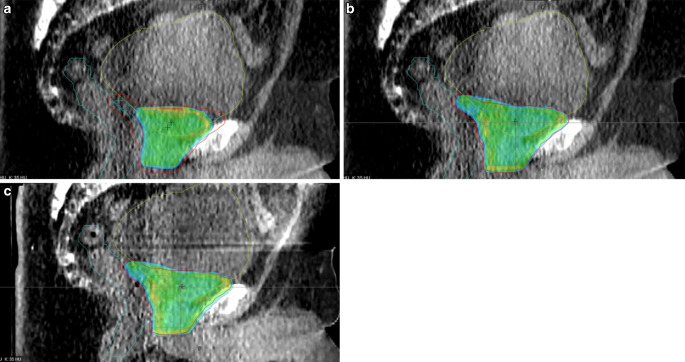
Visualization of the 95% isodose with colorwash towards 105% of the prescribed dose reveals **a** the scheduled plan (SCH) depicting a planning target volume (PTV, red) barely covered potentially due to fluctuations in bladder volume (yellow), while **b** the adapted plan (ADP) showcases a newly optimized dose distribution with improved coverage. **c** Dose reconstructed on the postadaptive verification CBCT

Figure 11 exemplifies how variations in nearby organ volume and shape (bladder) can markedly impact the dose distribution within and around the target. Figure 11a displays the original SCH plan, where the dose distribution barely encompassed the intended area (especially posterior to the bladder). In contrast, the ADP plan depicted in Fig. 11b demonstrates a reoptimized dose distribution. The verification plan with small differences compared to ADP due to time progression during adaptation is shown in Fig. 11c. During the specific treatment session shown in Fig. 11, the bladder volume observed was approximately three times larger than indicated in the initial pCT.

### Treatment time

The time required to align patients for conventional IGRT treatment (t_IGRTsetup_) was median 2:01 min (Q1: 1:39; Q3: 2:06); total time for IGRT (t_total, IGRT_) was median 7:53 min (Q1: 7:26; Q3: 9:12).

The time required for the adaptive process (t_adapt_) across all fractions in this study had a median of 15 min (Q1: 14; Q3: 18). In contrast, t_adapt_ for the initial treatment of each patient had a median of 22 min (Q1: 19; Q3: 22). The total treatment time, denoted as t_total, ART_, had a median of 25 min (Q1: 23; Q3: 27). As a result, t_total, IGRT_ was significantly lower than t_total, ART_ (*p* < 0.0001).

## Discussion

### Patient selection

Patients included in this study were primarily selected based on the availability of free capacity at the linear accelerator. This criterion allowed for a flexible approach to patient inclusion, while ensuring efficient utilization of treatment resources.

### Volume of organs at risk and targets

The volume of bladder and rectum varied extensively, which was made evident through oART. This may lead to the insight that many patients experience difficulties in presenting with comparably filled bladder and emptied rectum for daily radiotherapy sessions. This phenomenon was already described [12, 13]. Attempts were made by Yalman et al. [14] to establish patient-specific individual bladder-filling protocol to compensate for this effect, but this again depends on patient compliance. Especially the bladder volume of one patient was remarkable, as its median value was more than three times higher than on the initial pCT. Obviously, this initial image series was not acquired with filled bladder, as instructed. oART has the potential to address these challenges and alleviate the need for patients to adhere to instructions regarding bladder or rectum filling. Particularly oART could serve to offset any prominent anatomical variations observed solely on the day of pCT, mitigating potential impacts on the entirety of the treatment regimen, such as dosimetric inaccuracies stemming from minor CBCT alignment discrepancies or the need for additional imaging and replanning.

Equivalent to OARs, target volumes varied extensively during oART; a positive correlation between CTV volume and bladder volume was found. This could be explained by a larger bladder volume stretching the area of the prostate bed and thus enlarging the CTV volume. Again, one patient stands out with relative CTV and PTV volumes of about one and a half of its initial planning value. It is evident that this patient’s pCT was conducted with an empty bladder. Consequently, the increased filling level during treatment similarly affected the adjacent target volume.

### Dose to organs at risk and targets

Evaluation of dose to bladder and rectum shows that oART gives no or only little improvements in dose sparing in terms of D_mean_ or V_40%_ but may help to decrease dose variation (as described by interquartile range) during treatment. This effect was already described for other treatment sites in the pelvis region, e.g., for vulva [9].

Nearby bowel loops were taken into account by intended dose sparing which led to strong improvements on OAR protection but simultaneously to an inferior target coverage compared to the scheduled plans. This leads to the conclusion that such an adjustment of targets to highly variable organs should only be done if daily imaging and adaptation is available, as already described for MR-based online adaptive SBRT [15].

In general, the progression of time during the adaptation process leads to a deterioration in observed dose parameters and a widening of the IQR, though only to a very moderate extent.

Significant enhancements in target coverage were observed for both CTV and PTV, albeit less prominently for the former. This confirms the general concept of PTV margins. Additionally, the reproducibility of dose coverage, as indicated by the IQR, exhibited improvement what is also reflected in the increased clinical acceptability of ADP plans (and VER plans) compared to SCH plans and the fact that SCH plans were never chosen for treatment. Both enhanced coverage and improved reproducibility have also been described [16], specifically in the context of stereotactic treatment of the prostate. Figure 11a exemplifies how variations in nearby organ volume and shape (bladder) can markedly impact the dose distribution within the SCH plan. These variations may not be accounted for via IGRT and subsequent couch repositioning. In contrast, the ADP plan depicted in Fig. 11b demonstrates a reoptimized dose distribution. This revised plan considers the day’s anatomical variations and adjusts the dose distribution accordingly. The progression of time during adaptation leads to a moderate deterioration in dose distribution, as indicated by the verification plan shown in Fig. 11c. This underscores the importance of working time-efficiently during oART to avoid increasing deviations from the intended dose distribution. Nevertheless, the effects of anatomical changes during beam delivery, which can take several minutes, are still not accounted for in the VER dose and may require further investigation, especially since commercially available systems for intrafractional motion monitoring (e.g., surface-guidance or orthogonal X‑rays) are unable to detect geometrical variations in the soft tissue of the abdomen.

Again, patient 4 shows impressive characteristics as the improvement in D_98%_ of CTV and PTV were largest of all patients included in the analysis. This may again be explained by the large difference between bladder filling in the initial planning CT and the daily CBCT for reoptimization. It can consistently be concluded that especially patients with pronouncedly outlying characteristics, e.g., in terms of organ filling, benefit strongly from oART techniques, as the utilization of SCH plans would have resulted in substantial underdosing of the target area.

The common concept of using CTV–PTV margins seems to fulfill its object at least for interfractional variations, as even for the SCH plans, median D98% of the CTV was > 97% and so within the clinically acceptable range. Nevertheless, the use of ADP plans further increased this coverage and reduced outliers. This advantage is by no means negated by the effect of intrafractional variation, as shown by the verification plan. As oART does not necessarily include intrafractional monitoring, this should moreover not lead to the conclusion that PTV margins can be omitted completely. Nevertheless, a reduction of these margins, as suggested by Morgen et al. [17], should be investigated on in the future using larger prospective cohorts. A detailed analysis of the SCH dose distributions could also allow the derivation of anisotropic margins and should be correspondingly analyzed with a larger number of patients.

Further research is required to determine the extent to which enhanced dose coverage and reduced variation of dose exposure to OAR with oART, in comparison to IGRT, correlate with increased tumor control probability or decreased normal tissue complications probability.

### Time

The median total adaptation time averaged 25 min for oART treatments, but notably included several outliers, some exceeding one hour. This contrasts sharply with the rapid duration of median less than 8 min typically required for IGRT treatments, yet it still exceeds the in silico results of approximately 34 min described by Byrne et al. [18]. Moreover, in terms of time requirements, this approach is comparable to MR-based solutions [19, 20]. Consequently, oART necessitates more than triple the time of IGRT, involving additional personnel such as physicians and physicists on-site (legally mandated in Germany) and presenting a higher likelihood of unplanned delays due to increased variability.

These extended time requirements inherently constrain the treatment center’s capacities and scheduling flexibility for radiotherapy treatments. Consequently, careful patient selection becomes imperative, recognizing the challenging reality that offering oART to all patients may prove unfeasible within the current technological constraints. It becomes essential to prioritize and identify, through extensive long-term surveys, those patients who stand to benefit most from oART. It is also crucial to assess the suitability of oART for each individual patient, as inability to maintain position on the treatment couch, lack of understanding of the time-consuming adaptation processes during treatment, or claustrophobia can lead to extensive delays or even the cancellation of entire fractions [21]. This could ultimately negate the potential benefits of oART over IGRT.

With long time spans of 25 min for adaptive treatments, in some cases even more than one hour, and no intrafractional motion management, the need for a closing verification CBCT after adaptation and before irradiation becomes obvious. This time span may otherwise be long enough for highly versatile organs (e.g., bowel, bladder) to change the anatomical setting in a significant way. Even with shorter times for adaptation, there is a recognizable difference between the intended ADP dose and the VER dose. It is crucial to be aware of this circumstance to ensure accurate and effective treatment. While Xiong et al. [22] described the effects of intrafractional movement of the prostate as negligible for most cases [23], they found that omitting the verification CBCT led to larger margins being necessary to ensure sufficient dose coverage to the target. On the other hand, also the dose exposure to surrounding organs will probably be influenced: Bak et al. [9] described that in case of vulvar adaptive radiotherapy, the mean dose to bladder and rectum increased slightly in postadaptive verification CBCT, compared to the SCH as well as the ADP plan. This effect of duration of the adaptive process on the resulting dose distribution as well as required margins should be investigated further in the future.

## Conclusion

We demonstrated that implementing an online adaptive treatment approach for radiotherapy of the prostate bed resulted in enhanced coverage of the target volumes. While organs at risk did not exhibit significant dosimetric improvements, there was a reduction in the variability of data, as evidenced by a decrease in the interquartile range.

However, it is important to note that the time required for online adaptive workflows was found to be up to three times longer than conventional image guidance, necessitating increased staffing levels as well as more time at the treatment unit.

Moreover, the considerable time requirement for the adaptation process led to a subsequent degradation of dose distribution; however, this does not negate the overall advantage of adaptation. Efforts should be made to minimize the time needed for adaptation.

## Supplementary Information

The Supplementary Information provides a table containing the complete raw data set for all fractions analyzed in this study
